# Over 30 Years of DiI Use for Human Neuroanatomical Tract Tracing: A Scoping Review

**DOI:** 10.3390/biom14050536

**Published:** 2024-04-30

**Authors:** Georgios Mavrovounis, Aikaterini Skouroliakou, Ioannis Kalatzis, George Stranjalis, Theodosis Kalamatianos

**Affiliations:** 1 Department of Neurosurgery, School of Medicine, National and Kapodistrian University of Athens, Evangelismos Hospital, 10676 Athens, Greece; gmavrovounis@gmail.com (G.M.); stranjal@otenet.gr (G.S.); 2 Department of Biomedical Engineering, The University of West Attica, 12243 Athens, Greece; kskourol@uniwa.gr (A.S.); ikalatzis@uniwa.gr (I.K.); 3 Hellenic Centre for Neurosurgery Research “Professor Petros S. Kokkalis”, 10675 Athens, Greece; 4 Clinical and Experimental Neuroscience Research Group, Department of Neurosurgery, National and Kapodistrian University of Athens, 10675 Athens, Greece

**Keywords:** DiI, carbocyanine dyes, neuroanatomical tracing, axonal tracing, post-mortem human brain, neuroanatomy

## Abstract

In the present study, we conducted a scoping review to provide an overview of the existing literature on the carbocyanine dye DiI, in human neuroanatomical tract tracing. The PubMed, Scopus, and Web of Science databases were systematically searched. We identified 61 studies published during the last three decades. While studies incorporated specimens across human life from the embryonic stage onwards, the majority of studies focused on adult human tissue. Studies that utilized peripheral nervous system (PNS) tissue were a minority, with the majority of studies focusing on the central nervous system (CNS). The most common topic of interest in previous tract tracing investigations was the connectivity of the visual pathway. DiI crystals were more commonly applied. Nevertheless, several studies utilized DiI in a paste or dissolved form. The maximum tracing distance and tracing speed achieved was, respectively, 70 mm and 1 mm/h. We identified studies that focused on optimizing tracing efficacy by varying parameters such as fixation, incubation temperature, dye re-application, or the application of electric fields. Additional studies aimed at broadening the scope of DiI use by assessing the utility of archival tissue and compatibility of tissue clearing in DiI applications. A combination of DiI tracing and immunohistochemistry in double-labeling studies have been shown to provide the means for assessing connectivity of phenotypically defined human CNS and PNS neuronal populations.

## 1. Introduction

Anatomical tract tracing remains the gold standard for assessing white matter connectivity at the level of single axons. Over the past decades, driven predominantly by research on laboratory animals, neuroanatomical tract tracing techniques have evolved to allow for multi-dimensional paradigms assessing several anterograde (traveling in the neuronal cell body to axon direction) and retrograde (axon to cell body) tracers simultaneously and for the increased use of molecular–genetic tools, including viral tracers [1]. These invasive approaches, applied to numerous species from invertebrates to our closest relatives in the primates, continue to provide structural information that shapes fundamental aspects of our understanding of neural connectomics [2]. Recent efforts in the study of human structural connectivity have been spearheaded by the explosion of diffusion magnetic resonance imaging (dMRI) tractography studies including those on large-scale cohorts such as in the Human Connectome Project [3]. Despite significant advances in computational techniques [4], tractography results can nevertheless lead to questionable interpretations due to several known limitations associated with their resolution and the spatial orientation of primarily smaller, crossing, kissing, and bending fibers [5]. In this context, validation of human structural neuroimaging data, especially in cases of novel or lesser-studied tracts, remains a significant consideration. Nevertheless, obtaining human “ground truth” connectivity data in a manner analogous to animal tract tracing studies [2] can only be approached by a limited number of ex vivo techniques. In recent years, one such technique, the gross white matter dissection method first developed by Joseph Klingler in the 1930s [6,7], has made a comeback not only as an educational but also as a valuable research tool clarifying the topographic anatomy of several human white matter tracts [8,9,10]. While ideal for investigating relatively long/large fiber bundles, Klingler’s technique has nevertheless limited utility for smaller bundles that pass through areas of dense crossing and for establishing axonal terminations [11], other than those at the outer limits of the cerebrum.

The more recently introduced polarized light imaging and polarization-sensitive optical coherence tomography techniques provide a superior level of resolution compared to Klingler’s technique that is nevertheless over the level of single axons, at 10s-100s µm spatial resolution [11]. These techniques rely on the optical property of materials termed birefringence to visualize structures with different optic axis orientations [11] and have been applied to study human grey and white matter architecture [12,13,14,15].

Ex vivo human tract tracing techniques supporting axonal level microscopic analysis are very limited. The most commonly used method is the one utilizing long-chain lipophilic carbocyanine dyes [16,17]. Carbocyanine dyes are a series of fluorescent dyes with different spectra of absorption/emission that can be readily incorporated in the lipid bilayer of cell membranes and diffuse freely along the membrane, due to their lipid solubility [18]. They are not soluble in water, yet their fluorescence becomes easily observable once they are integrated into membranes [19]. Even when used in aldehyde-fixed tissues, they proceed to travel bidirectionally, that is both anterogradely and retrogradely, by diffusion [20]. The capability for both retrograde and anterograde labeling to occur at the same time can either benefit or hinder the aims of a study. For example, bidirectional labeling is advantageous for identifying the origins of fibers that intersect at the dye application area, providing a clearer understanding of neural pathways [18]. The most commonly applied carbocyanine dye in post-mortem human studies is DiI or 1,1-dioctadecyl-3,3,3′,3′-tetramethylindocarbocyanine perchlorate, which achieves labeling by inserting two long hydrocarbon chains into the lipid bilayer and emits red fluorescence at 565 nm [16]; Di-O is another less-frequently-used dye. Various analogs of the aforementioned dyes have been developed and used in other tissues/animals that offer certain advantages, like avoiding autofluorescence and allowing two-color labeling (DiD, red-shifted excitation/emission spectra), easier incorporation into membranes [DiIC_12_(3), DiIC_16_(3) are less lipophilic], and accelerated diffusion (Fast DiI, Fast Di-O) [21]. While offering a means of studying human white matter connectivity at the unsurpassed (microscopic) level, previously indicated disadvantages of lipophilic tracers include the bidirectional nature of tracing, extremely long dye incubation periods, and short tracing distances [16,22]. Another limitation of DiI that has been observed in experiments utilizing animal tissue including embryonic tissue, is off-target (non-specific) labeling due to the transneuronal transfer of the dye. It happens when DiI is transferred to a second-order neuron or other nearby fibers [20,23]. An additional mechanism for off-target (non-specific) labeling results from the dispersion of DiI crystals within the incubation solution [17]. 

The aim of the current scoping review is to map and present the available data in the literature regarding the use of DiI for neural tract tracing in the human nervous system.

## 2. Materials and Methods

The protocol for the present scoping review was written according to the Preferred Reporting Items for Systematic Reviews and Meta-Analyses Extension for Scoping Reviews (PRISMA-ScR) [24,25], and it was published online on Open Science Framework (OSF) Registries before the initiation of the study. The full protocol is available in full at Registration doi:10.17605/OSF.IO/Y3BCT.

### 2.1. Literature Search

Two independent authors (G.M., T.K.) performed an electronic search of the PubMed, Scopus, and Web of Science databases. The implemented algorithms included the following search terms and their synonyms and variations combined with the Boolean operators “OR” and “AND”: DiI, neuron, nerve, axon, brain, spine, plexus, trace. The exact algorithms applied in all databases are available in Appendix A Table A1. The last literature search was performed on 18 March 2024. To identify additional studies, the authors manually reviewed the bibliography lists of the studies that fulfilled the inclusion criteria.

Following automated duplicate removal, the titles and abstracts of all identified studies were reviewed to choose potentially relevant studies. Subsequently, the full texts of potentially relevant studies were reviewed and tested against our inclusion and exclusion criteria to decide on the included studies. All disagreements were resolved by a third member of the team (G.S.).

### 2.2. Study Selection

The following inclusion and exclusion criteria were applied for study selection: We included (1) laboratory-based studies/reports (2) using the lipophilic tracer DiI and/or other carbocyanine dyes (3) for axonal tracing (4) in human (5) central nervous system (CNS) and peripheral nervous system (PNS). We only included studies (6) in the English language. (7) No restrictions were applied on publication dates. We elected to focus mainly on DiI, as it has been reported to be the most commonly used carbocyanine dye and the one with the fastest tissue diffusion time [16,17].

We excluded (1) narrative and systematic reviews of the literature, (2) letters to the editor and short communications without original data, (3) cell culture-based and animal studies, and (4) conference abstracts.

### 2.3. Data Extraction

Two independent authors (G.M., T.K.) performed the data extraction process using identical Excel forms. The following data, when available, were extracted from all studies: first author’s name, year of publication, study objectives, type of specimen used (e.g., brain, spinal cord, cranial or peripheral nerve, peripheral organ, etc.), methodologies for fixation and staining, pathways traced, and tracing distances achieved, as well as additional cellular components stained (e.g., cell bodies, dendrites/dendritic spines). 

## 3. Results

### 3.1. Study Selection

**Figure 1:**
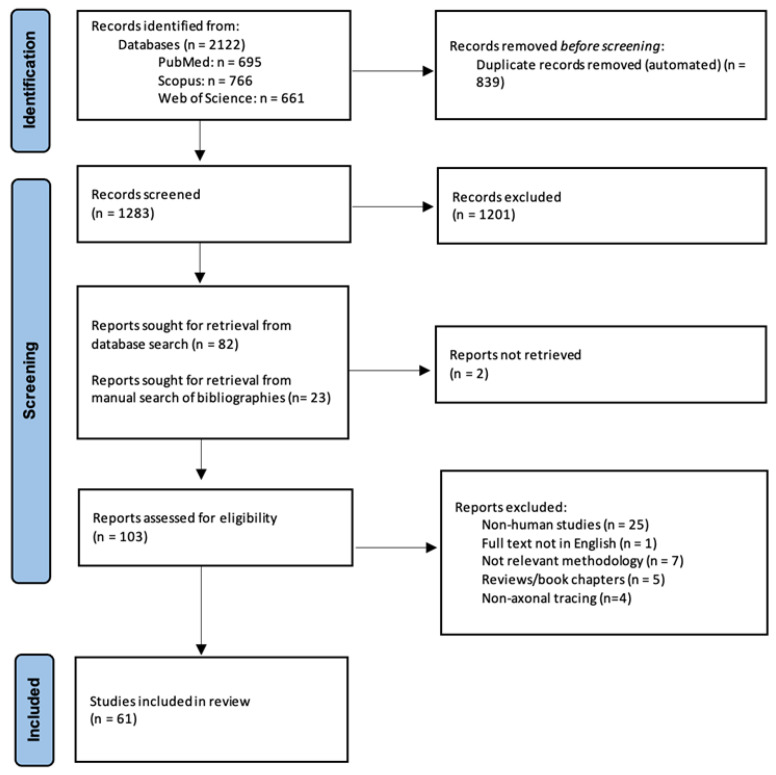
PRISMA-ScR flowchart for study selection.

A flow chart on the study selection process is given in Figure 1. The literature search resulted in 2122 articles. After an automated removal of duplicates, 1283 articles remained. Following title and abstract review, 1201 manuscripts focusing exclusively on animal research were excluded. Following full text reading of the remaining 82 studies, 38 studies fulfilling inclusion criteria were identified. Twenty-three additional studies were identified through a manual search of the reference lists of included articles; the full texts of two studies could not be retrieved and, thus, were excluded. The reasons for exclusion for the remaining studies were as follows: non-human studies (n = 25), full text not in the English language (n = 1), not relevant methodology (n = 7), reviews/book chapters (n = 5), non-axonal tracing (n = 4). Overall, 61 studies, using mainly DiI for axonal tracing, were included in the present scoping review. 

### 3.2. Classification of Included Studies 

Table 1 presents the extracted data in terms of the first author’s name/year of publication, main study objectives, specimen types utilized, methodological considerations, and tracing distances achieved. The 61 included studies [17,22,26,27,28,29,30,31,32,33,34,35,36,37,38,39,40,41,42,43,44,45,46,47,48,49,50,51,52,53,54,55,56,57,58,59,60,61,62,63,64,65,66,67,68,69,70,71,72,73,74,75,76,77,78,79,80,81,82,83,84] were published during a 33-year period, between 1989 and 2022. 

Overall, brain tissue specimens were utilized by the majority of studies (n = 40). In addition, 14 studies utilized peripheral organs (gastrointestinal system, eyes/retinas) and a smaller number of studies utilized spinal cords (n = 3). Some studies utilized more than one type of specimen. While most studies (n = 36) used adult specimens, a significant number of studies (n = 27) incorporated specimens at various developmental stages from the embryonic period to childhood, adolescence, and adulthood; one study did not provide age information regarding its specimens. A trend for the inclusion of higher numbers of subjects (average n = 14), was apparent for studies utilizing peripheral organs for DiI tracing. 

The included studies covered a wide variety of anatomic regions and pathways. The visual pathway, including the retina, the optic nerve, the optic tract, the optic radiation, the superior colliculus, and the visual cortex, was the most commonly studied pathway (n = 14 studies). Three studies explored connections within the visual cortex and their development. Another three studies investigated the morphology of retinal ganglion cells, while two studies looked into the retinotopic fiber organization. The remaining studies investigated the retinofugal fibers to the pretectal region (n = 1), the retinohypothalamic tract (n = 1), the retinogeniculate, thalamocortical (n = 1) and retinocollicular (n = 1) connections, the intercollicular pathway (n = 1), and the intrinsic connections of the superior colliculus (n = 1). 

A substantial number of studies (n = 7) focused on the hippocampal formation, the entorhinal cortex, and other structures of the limbic system (amygdala, mammillary bodies). Four studies investigated the cortico-cortical connections within the medial temporal lobe and the hippocampal formation, such as the alvear, perforant, and endofolial pathways, the Schaffer collateral system, and temporo-entorhinal connections. The remaining studies investigated pre- and post-synaptic connections of the hippocampal Cornu Ammonis 1 (CA1) region, the amygdala, and the hypothalamic connections to the limbic structures. 

Similarly, nine studies investigated the nerve plexuses of the gastrointestinal tract, focusing on the innervation of the pylorus (n = 1) and the motor and non-motor neurons of the small and large intestines (n = 8). Other areas of interest included the upper (n = 4) and lower (n = 4) cranial nerves and their nuclei, various subcortical and non-cranial nerve brainstem nuclei (n = 5), the auditory cortex (n = 3), and the spinal cord and peripheral nerves (n = 5). 

### 3.3. Unique Applications

One study [69] utilized DiI tracing to study the innervation of the middle cranial fossa’s dura mater, reporting that collateral branches of the meningeal afferents of the mandibular nerve innervated extracranial tissues. The authors suggested that this innervation should be studied further as it is possibly involved in the pathogenesis of headaches. Another notable application was performed by Onodera et al. [61], who used DiI to identify the borders of the aged human red nucleus and the rubrospinal tracts, suggesting that the magnocellular red nucleus of adults is smaller and rotated when compared with that of infants. Interestingly, one study [70] implemented DiI tracing to validate the findings of ex vivo dMRI, reporting good correspondence between the two techniques in a temporal lobe tissue block. 

A group from Germany published two studies [27,28] investigating fiber orientation in the human internal capsule, utilizing a combination of polarized light and confocal laser microscopy, the latter for DiI detection. In their first study, they determined that anterior capsulotomy (lesion of the anterior thalamic peduncle) for obsessive-compulsive disorder works by disconnecting the fronto-basal-thalamic loops [28]. Furthermore, they proposed a parcellation system of the internal capsule using 3D fiber orientation as the criterion [27]. 

Finally, it is worth mentioning that in addition to axonal tracing, several studies utilized DiI to investigate normal and pathological neuronal features such as those of dendrites/dendritic spines, axons, and cell body morphology. Some notable applications include the investigation of the shape and size of the somata of sympathetic preganglionic neurons and motoneurons of the spinal cord [53], the investigation of dendritic abnormalities in patients with Rett syndrome [29] and argylophilic grain disease [77], and the investigation of developing neuronal characteristics (e.g., spines, axons, cell orientation) in various nervous system components (e.g., substantia nigra, visual cortex, corpus callosum) and various pre- and postnatal ages [37,63,68]. 

## 4. Discussion

### 4.1. Overview

The present scoping review aimed at mapping the available literature on the use of the carbocyanine dye DiI for neuronal tracing in human specimens. We identified 61 studies, spanning four decades, using different forms of DiI to study neuronal projections within the central and peripheral nervous systems. 

### 4.2. Initial Application

In 1989, Burkhalter et al. published the first human study using DiI crystals in paraformaldehyde-fixed occipital lobe specimens to investigate the cortico-cortical connections of the visual cortex [32]. The authors reported that the findings in the fixed brain tissues resembled those of in vivo axonal tracing in non-human primates. Consequently, they concluded that DiI tracing could prove useful in studying age- or disease-related structural alterations in human post-mortem brain specimens and enable the study of human-specific brain regions. Indeed, since its initial application, DiI has been utilized for tract tracing in several studies investigating both PNS and CNS targets such as such as the optic pathway, the hippocampus, the spinal cord, peripheral nerves, and nerve plexuses [17,40,57,59,79,80].

### 4.3. Protocols for Carbocyanine Tract Tracing

#### 4.3.1. Basic Methodological Steps

**Figure 2:**
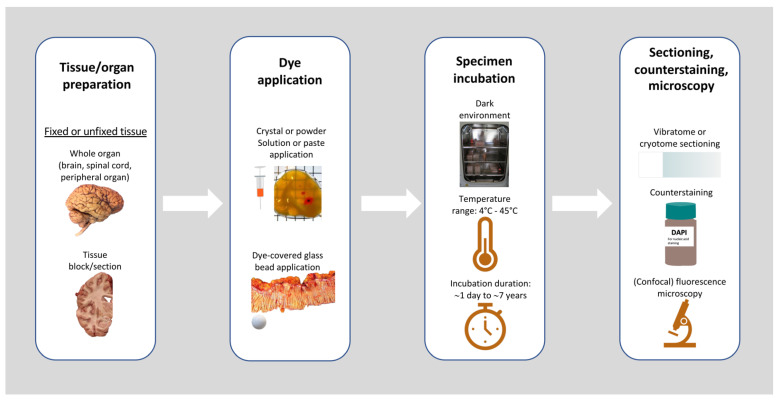
Basic methodological steps and their variations reported in previous studies. Regarding tissue preparation, fixed or unfixed tissues have been used in the form of whole organs or tissue blocks/sections. Various modes of dye application have been utilized ranging from crystal/powder to solution/paste and dye-covered glass beads. Incubation of specimens, preferably in a dark environment, has been reported to take place at temperatures ranging between 4 and 45 °C. The duration of incubation also varied widely between a few hours and years. After incubation, the specimens were sectioned and DiI was observed using fluorescence microscopy in the presence or absence of counterstaining. [Images used/adapted with permission (Creative Commons CC-BY license): [47,85,86,87].

Various methodologies have been described for neuroanatomical carbocyanine dye tracing in human specimens. Nevertheless, most protocols include the following four basic steps. Figure 2 illustrates the basic methodological steps for DiI tracing.

Step 1: Tissue preparation for dye application

Initially, whole specimens [37] and thick [32] or thin [71] slabs of post-mortem tissue undergo fixation in aldehyde fixatives. The most commonly used fixative in the included manuscripts (Table 1) was 4% paraformaldehyde (PFA). Some studies have used a sequential fixation process, initially immersing tissue in formalin followed by a subsequent immersion in PFA [30]. Importantly, some authors delayed the fixation process until after DiI application (post-fixation) to enhance diffusion distances (see below) by avoiding the crosslinking of membrane proteins caused by aldehyde fixatives [22]. 

The duration of fixation varies from study to study, ranging from a few hours to several years [35,37,54,61,62]. In the study by Lukas et al., one of the few studies that systematically assessed the influence of several parameters including the nature of fixation on tracing efficacy [17], an initial perfusion of postmortem cadavers with a lower PFA concentration (0.5%) resulted in shorter tracing distances. Some authors reported that an initial fixation of brain tissue with 10% formalin resulted in suboptimal staining [40], with defective neuronal transport, albeit preserved glial staining [46]. Nevertheless, of note, several studies indicate the use of formalin-fixed tissue at concentrations of 2% [26], 10% [30,55,57,61,72], 20% [51], or 4% for long-term storage, the latter for fetal archival tissue [62].

A postmortem delay in fresh tissue fixation prior to dye application has been indicated as an impeding factor for DiI tracing [17,52].

Step 2: Dye application

Several methods for dye application are described in the literature. For the current scoping review, we sorted the techniques for DiI application (similar techniques are used for the rest of the carbocyanine dyes) into three main categories, namely DiI crystal/powder, DiI solution/paste, and DiI-coated glass beads. The most commonly used method identified in the present review was crystal application directly into the tissue using glass micropipettes, stainless steel wire, or needles [32,42,45]. One study used fine crystal powder that was directly applied onto the specimens [61]. When using crystals, previous studies indicate the need for caution in order to avoid contamination of specimens with crystals in areas other than the target region, as this could result in false positives. Failure to trace fetal root afferent projections when DiI crystals were placed on the dorsal root or the dorsal ganglion due to the long distance between the site of placement and the spinal cord was indicated by Konstantinidou et al. [52]. 

Another form of DiI that can be used is DiI solution. Typically, ethanol is used as the solvent and a small amount of the resultant solution is injected into the target area [56]. Solution drops can also be applied directly onto sections that are already placed on microscope slides [27,28] or nerve stumps can be directly placed into the solution for tracing that involves specific nerves [48]. Similarly, pastes (crystals and alcohol) can be applied directly on the tissues under study [60,62]. Notably, Sivukhina et al. utilized injections of DiI dissolved in 100% ethanol to human amygdala sections and reported diffusion speeds of approximately 0.1 mm/day, indicating that DiI tracing can be achieved in the course of days/weeks instead of months [71]. It is important to mention that Axer et al. [27] have used the analog Fast-DiI dissolved in dimethylsulfoxide to study the internal capsule. 

Finally, a different technique has been used for the labeling of neuron plexuses in the gastrointestinal tract. In these instances, an alcoholic dye solution is allowed to dry onto glass beads that are then pressed directly into the tissue under study [64,79,80].

Step 3: Dye incubation

Following dye application, the specimens should be incubated in the dark to allow for dye diffusion. Generally, the specimens are stored in a solution similar to the one used for fixation [35,37,38] but other solutions like phosphate buffer have also been widely used [32,33,52]. In fixed animal tissues, ethylenediaminetetraacetic acid (EDTA) has been shown to bind calcium and minimize dye leakage and off-target (non-specific) tracing [88]. It is known that calcium precipitates lipids in membranes and can interact with lipids leading to the production of insoluble compounds. Dyes incorporated in these compounds might be able to travel between membranes, thus producing off-target (non-specific) tracing. 

Significant differences between studies are apparent regarding incubation times and temperatures. Most studies used incubation periods ranging from one week to one year [27,29,30,35,45,55,73], while some researchers extended their incubation periods for more than a year [17,70,76], reaching seven years in one study [61]. Regarding the temperature of incubation, the included studies used mostly room temperature [37,45,61], 37 °C [62,76], or 4 °C [48,63]. Lukas et al. reported that an incubation at 37 °C (but not 40 °C) for a period of 12–15 weeks is optimal [17]. At these conditions, a dye diffusion speed of approximately 0.01 mm/h, using DiI crystals applied to the human cervical spinal cord, was achieved [17]. During prolonged incubation periods (over 15 weeks) crystal displacement and dispersion within the incubation solution can result in off-target (non-specific) tracing. In longer incubation periods, DiI fading has also been observed [17].

Step 4: Tissue preparation/sectioning, counterstaining, and microscopy

Prior to mounting onto glass slides for microscopic investigation, sectioning of the tissue under study is typically performed using a vibratome (tissues usually embedded into agar [35,42,52]) or a cryostat/freezing microtome following cryoprotection with sucrose solutions and freezing of the specimens [17,18,46]. Notably, cryostat sectioning has been reported to cause dye leakage [17]. The sections (thickness range: 10 μm–350 μm, some studies used whole-mounted tissues [78]) are then mounted onto adhesive (e.g., gelatin-coated) [17] or uncoated [84] glass slides. In fixed animal tissue, phosphate-buffered saline (PBS) has been identified as a superior mounting medium when compared to glycerol with n-propyl gallate, which exhibited more dye leakage and off-target (non-specific) labeling, as well as decreased clarity [88]. Fluorescence (including confocal) microscopy with rhodamine filters allows for DiI visualization and analysis [17,18]. Further anatomical delineation can be achieved using several counterstains (on DiI-stained or alternate sections), such as propidium iodide [30], bisbenzimide [42], DAPI [37,45,46,82,83,84], Nissl stain [37,52,57,73,74,75,76], acridine orange [56]. The use of anti-fade mounting media can prevent or minimize fluorescence quenching [22,69].

#### 4.3.2. Gastrointestinal Tract Plexuses: Methodological Considerations

The methodological approach followed by most researchers studying the gastrointestinal tract nerve plexuses differs from the rest of the studies. All but one of the studies used surgical specimens from patients undergoing operations for either biliary atresia [44] or cancer [49,50,64,65,66,79,80]. 

All but one study [44] followed similar protocols. In brief, after resection, the specimens were placed into (modified) Kreb’s solution for transportation to the lab. The tissues were then prepared for dye delivery and DiI-covered glass beads were placed into the areas of interest. Specimens were then stored in organotypic culture medium in a humidified incubator for three to five days to allow for dye diffusion, and then fixed in Zamboni’s fixative for periods ranging from 16–48 h at 4 °C. Finally, they were whole-mounted and studied with fluorescence microscopy [66].

### 4.4. Investigations Aimed at Optimizing or Broadening the Scope of Carbocyanine Tract Tracing 

Several previous studies aimed at optimizing the various methodological steps discussed in the previous paragraphs or broadening the use of DiI applications on human specimens. Pertinent approaches are discussed below. 

#### 4.4.1. Use of Archival Specimens

Another important factor limiting the use of carbocyanine dyes in human specimens is the fact that acquiring recently-fixed post-mortem nerve tissues for research purposes is often difficult and expensive [62]. Due to the lipophilic nature of carbocyanine dyes, most protocols developed for their application in human tissues were based on specimens that had been paraformaldehyde-fixed for no longer than a year, to ensure maximal lipid preservation. Consequently, studies on archival tissue were thought to be unfeasible [62]. Nevertheless, a recent study applied DiI, DiO, and DiD crystals and pastes on various archival neural tissues, specifically the thoracic dorsal root ganglia, and the facial, sciatic, tibial, vagus, and vestibulocochlear nerves of embryos that were immersion-fixed in 10% formalin for a mean time of 16.75 years [62]. The authors indicated similar tracing distances to those in previous studies and concluded that archival tissue is suitable for neuroanatomical tracing [62]. 

#### 4.4.2. Tracer Re-application 

Results on the utility of dye re-application once or more throughout the incubation period to optimize labeling have been contradictory [17,56]. Loelinger et al., in their study of lower cranial nerves in post-mortem brainstems of infants, reported that they observed optimal staining when they re-applied the DiI solution three to four months after initial application (for a total incubation period of at least eight months) [56]. Of note, Lukas et al. did not observe any noticeable improvement in diffusion distances with DiI crystal re-application in their study of post-mortem adult spinal cords and peripheral nerves [17]. 

#### 4.4.3. Delayed-Fixation Method

The main limitations of DiI neuronal tracing techniques are the small tracing distances from the injection/application site and the long incubation periods needed for dye diffusion [22]. Hypothesizing that the aforementioned limitations were mainly due to the effects of aldehyde fixation on the molecular structure of nervous tissues, Sparks et al. suggested a “delayed fixation” method, previously used in animal studies [89], to enhance and accelerate DiI diffusion in human brain specimens. In short, they injected the Fast DiI solution to human brain samples within 3 h post-mortem and incubated them for 36 h at 4 °C. The samples were subsequently fixed in 4% buffered paraformaldehyde for 5 days. The authors observed tracing distances of 20–40 mm and thus a diffusion speed of nearly 1 mm/h. In conclusion, they suggested that the delayed-fixation method provides faster (within a week) and more effective Fast DiI tracing results. A similar methodology was used by Hufner et al. in successfully studying the ophthalmic branch of the trigeminal nerve [48].

#### 4.4.4. Application of Electrical Fields

An interesting approach to increasing the diffusion rate and diffusion distance of carbocyanine dye tracing in human nerve tissue was taken by Swift et al. [72]. Considering the cationic nature of the dyes in combination with data from studies on individual neurons indicating that DiI moves when a voltage gradient is present [90], the authors applied direct electric current (10–40 V/cm) to peripheral nerve specimens after carbocyanine solution application. They reported that in the specimens to which an electric current was applied (field-enhanced diffusion), the diffusion rate was increased by 100 times to approximately 1 mm/h.

#### 4.4.5. Photoconversion of Labeled Tissues

The fluorescent signal of the carbocyanine dyes is not permanent and will photobleach with prolonged exposure [18]. This can be in part ameliorated with the use of photoconversion. This technique uses diaminobenzidine (DAB) and light exposure through the epifluorescence microscope, after dye application and diffusion, to start a redox reaction, resulting in a stable brown product within 30 to 90 min [18,38,52,57]. Notable drawbacks of the technique include its time-consuming nature, that it should be performed as soon as possible after tissue sectioning, and the potential for axonal breakage [18].

#### 4.4.6. Optical Clearing in DiI Tracing Applications 

Tissue-clearing techniques aim at minimizing lateral scattering of light, rendering biological specimens transparent and enabling the in-depth study of intact tissues or even whole organs [91]. A wide variety of techniques have been described and can be broadly categorized into the following: aqueous-based tissue clearing, organic solvent-based tissue clearing, and tissue clearing based on hydrogel embedding [91,92]. The combination of lipophilic tracing with optical tissue clearing could aid in studying region-specific microcircuitry [47]. 

In 2018, Lai et al. reported the results of their efforts to combine modern tissue-clearing techniques with conventional non-immunohistochemical staining methods, such as DiI tracing and cresyl violet staining, in both fresh and archival human brain specimens [54]. For DiI tracing, they used OPTIClear, a refractive index homogenizing agent, which is detergent- and denaturant-free, making it appropriate for lipophilic tracing. Interestingly, they succeeded in tracing mossy fibers with dendritic spine-like projections from the deep white matter up to the granular layer in a single cerebellar folium. The main limitation reported for the techniques used in this study, true for most tissue-clearing applications in human specimens [93], was the inherent difficulty of cleared thick human tissue blocks to be effectively combined with immunohistochemistry. Hildebrand et al. also developed a tissue-clearing protocol for fresh brain samples that can be combined with lipophilic tracers [47]. Using the developed approach, they succeeded in identifying dendrites with spine-like protrusions in brain samples containing the amygdala. It is notable that they reported superior clearing results with the use of Periodate-Lysine-Paraformaldehyde fixative, instead of formalin, for 2–7 days prior to labeling [47].

#### 4.4.7. Double-Labeling Methods: Combination of DiI with Immunohistochemistry

The combination of DiI tracing with immunohistochemistry (ICH) in double-labeling experiments, offers a significant advantage over single DiI tracing, namely the identification of the neurochemical phenotype of neurons projecting to specific central or peripheral sites. The most frequent application of this double-labeling method identified in the present review concerned the neurochemical characterization of neurons of the human colon [50,64,65,66,80]. The incompatibility of DiI tracing with Triton X-100, a detergent typically utilized to improve tissue penetration of antibodies in immunohistochemical applications, was indicated in a double-labeling study in the human small intestine [44]. DiI tracing with ICH has also been recently used in the human brain to determine the peptidergic phenotype of hypothalamic neurons projecting to the distinct limbic system targets [71]. 

#### 4.4.8. Particle-Mediated Labeling

During our database search, we identified studies implementing particle-mediated labeling using DiI. This technique, referred to as DiOlistics, involves staining tissue sections by using DiI coated onto tungsten particles, which are then delivered onto the sections using a gene gun. Data were not extracted for these studies as they did not fulfill our inclusion criterion of “tract tracing” [94,95,96]; they used DiI to study characteristics of dendrites in sections. 

### 4.5. Limitations of the Present Review

The limitations of our current study were mainly those inherent to most scoping reviews of the literature [97]. The possibility of missing relevant studies due to the exclusion of conference abstracts and studies not written in English should always be kept in mind. Furthermore, although we included studies identified in three of the largest scientific databases, studies not indexed in those were unavoidably excluded. It should be mentioned that in order to minimize the possibility of excluding relevant studies, we manually reviewed the bibliographies of all the included studies to identify articles fulfilling our criteria. 

## 5. Conclusions

Over the course of the last three decades, a relatively small number of studies have utilized the fluorescent tracer DiI to assess human neuronal connectivity at the microscopic level. Among them, a limited number of studies have systematically addressed the optimization of the technique to improve efficacy. While DiI neuroanatomical tracing has been applied to assess nervous system development, most studies focused on adult tissue. The majority of identified studies focussed on the central nervous system. The connectivity of the visual pathway was the most commonly studied topic. DiI crystals were the most commonly applied form, with a smaller number of studies utilizing paste or solvent-dissolved dye. The maximum tracing distance and tracing speed achieved in previous studies were 70 mm and 1 mm/h, respectively. We identified studies that focused on optimizing tracing efficacy by varying parameters such as specimen fixation, incubation temperature, dye re-application, or the application of electric fields. Additional studies aimed at broadening the scope of DiI use by assessing the utility of archival tissue and compatibility of tissue-clearing methods in DiI tracing applications. The possibility of converting DiI into a permanent product was addressed by studies utilizing photoconversion. A combination of DiI tracing and immunohistochemistry in double-labeling studies have been shown to provide the means for assessing the connectivity of phenotypically defined human neuronal populations. 

## Figures and Tables

**Table 1:** Table presenting the main characteristics of the included studies.

First Author YOP	Specimen Type, N of Subjects (DiI Tracing)	Specimen Fixation, Processing for Tracer Application	Application Method	Application Site	Tracing Distance (Max)	Incubation Solution Time Temperature	Study Objective
Axer H. 2000 [27]	Brain, 4	Collected brains fixed in 4% aqueous formalin solution, sucrose cryoprotected, 60 µm sections	Solution covering (3–4 drops of 1 mg DiI in 1100 µL DMSO)	Internal capsule sections	NA	DMSO 7 days 37 °C	Fiber location and orientation of the internal capsule in adults
Axer H. 1999 [28]	Brain, 4	Collected brains fixed in 4% aqueous formalin solution, sucrose cryoprotected, 60 µm sections	Solution covering (3–4 drops of 1 mg DiI in 1100 µL DMSO)	Internal capsule sections	NA	DMSO 7 days 37 °C	Fiber structure in the anterior limb of internal capsule in adults
Belichenko P. 1994 [29]	Brain, 1	Tissue blocks following 1 month formaldehyde fixation	Crystal placement	White matter or layers I and IV of cortex	NA	4% PFA 2 to 6 months NA	Effects of Rett syndrome on cortical architecture and afferent axons, assessed in a 16-year-old and 2 adult patients in comparison to normal subjects and patients with epilepsy
Bose S. 2005 [30]	Brain and optic nerve, 2	Tissue initially fixed in 10% formalin, followed by 2% PFA and 2% glutaraldehyde for 1–3 weeks	Crystal placement	Brachium of the superior colliculus	NA	NA 4 weeks NA	Pupillary fibers of the pretectal region of an adult with LOHN compared to a healthy control
Burkhalter A. 1989 [32]	Brain, 4	Tissue blocks fixed in 3% PFA solution containing 0.1 M lysine-HCI, 0.8% NaIO4, and 0.8% iodoacetic acid for 24 h at 4 °C	Crystal placement	V1 cortex	6 mm	Phosphate buffer 2 to 8 weeks 21 °C	Circuitry of visual cortex within the occipital lobe in adults
Fitzgibbon T. 1996 [39]	Eye, 32 retinae/NA *	Fixed in 2–4% PFA	Crystal placement	Nerve fiber layer of macula region or various retinal locations	8–10 mm (estimate)	2–4% PFA 4–24 weeks 37 °C or room temperature	Development of connections in the human visual cortex at various gestational ages
Friedman D. 1991 [40]	Brain and optic nerve, 6	Tissue blocks fixed in 4% PFA	Crystal placement	Distal end of optic nerve	10 mm	4% PFA 3–4 months 37 °C	Labeling of the human retinohypothalamic tract in adults
Hannan A. 1999 [42]	Brain, 4	Formalin-fixed tissue blocks	Crystal placement	Vicinity of heterotopic nodules	2–3 mm	4% PFA 3–6 months 37 °C	Connectivity of heterotopic nodules in children
Hufner K. 2009 [48]	Trigeminal ganglion/opthalmic nerve, 11	Delayed fixation using 4% PFA	Solution covering (17% Fast DiI in dimethylformamide)	Nerve stump	5 mm (estimate)	Dimethylformamide 10–14 days 4 °C	HSV-1 infiltration of the trigeminal ganglion and nerve in adults
Kakita A. 2002 [51]	Brain, 1	Specimens in 20% formalin	Crystal placement	Periventricular nodule surface or adjacent white matter	2–3 mm (estimate)	NA 5 months 37 °C	Effect of bilateral periventricular nodular heterotopia due to filamin 1 gene mutation on characteristics and connectivity of nodules in an adult patient
Krassioukov A. 1998 [53]	Spinal cord, 1	Spinal cord fixed in 4% formalin (2 weeks)	Solution injections (80–100 μL of 4% DiI solution in 100% ethanol)	Ventral portion of spinal segments, ventral root origin	NA	Phosphate buffer 7 months 4 °C	Method for retrograde labeling of preganglionic neurons and motor-neurons in human thoracic spinal cord in adults
Lim C. 1997 [55]	Brain, 6	10% formalin-fixed brains (>2 weeks), 1 mm thick slabs, tissue blocks	Crystal placement or Solution injections (45 nL of 1% solution DiI in 100% ethanol)	Fields and layers of hippocampal formation	8 mm	Formalin up to 1 year 37–45 °C	Connections of pyramidal neurons of hippocampal regions in adults
Lukas J.R. 1998 [17]	Spinal cord, sciatic nerve, brachial plexus, 6	4% PFA or 4% carbol and 0.5% PFA perfusion-fixed cadavers	Crystal placement	Proximodistal direction to sciatic nerves and to brachial plexus or the lateral or posterior funiculus or dorsal columns of spinal cord	28.5 ± 2.3 mm (12–15 weeks)	4% PFA up to >1 year 37 °C or 40 °C	Methods for optimizing tracing in adult tissue
Mufson E. 1990 [59]	Brain, 1	Coronal sections (1 cm) fixed in 4% PFA for 24 h at 4 °C	Crystal placement	Separate subregions of hippocampal complex	8 mm	4% PFA 6 months 20 °C	Validation of DiI as a neuronal tracer in fixed brain in adults
Onodera S. 2010 [61]	Brain, 7 **	10% formalin solution-fixed cadaver, tissue blocks	DiI powder	Dorsomedial, ventrolateral parts of the rostral red nucleus or the ventro/dorso-lateral zones of the caudal red nucleus	10 mm (estimate)	4% PFA 7 years room temperature	Morphology and boundaries of the red nucleus in adults
Schueler M. 2014 [69]	Brain with dura (to use some nerve branches), 3	Unfixed skull, nerve stumps	Crystal placement	Distal nerve stump	NA	4% PFA 6–7 months 37 °C	Dura mater and extracranial tissue innervation in adults
Sivukhina E. 2020 [71]	Brain, 6	Tissue sections (6–10 mm), immersion-fixed in 4% phosphate buffered PFA	Solution injection (1–2 μL of 1 mg/mL DiI in 100% ethanol)	Amygdalar nuclei	20–30 mm	4% PFA approx. 10 days room temperature	Hypothalamic projections from oxytocin and arginine-vasopressin neurons to limbic system targets in adults
Sparks DL. 2000 [22]	Brain, 1	Delayed fixation: fixation in 4% PFA delayed until 36 h following DiI application	Solution injection (5 μL of 1.7 mg/mL Fast DiI in dimethylformamide)	Lateral Cerebellum	20–40 mm	NA 36 h NA	Method of delayed-fixation for tracer diffusion in adult brain
Tardif E. 2001 [73]	Brain, 4	4% PFA perfused brain and 12 h postfixation in same fixative	Solution injection (0.1– 0.2 μL of 10% DiI in dimethylformamide)	Parts of auditory cortex	5 mm	4% PFA 6–12 months NA	Intrinsic connectivity of auditory areas in adults
Tardif E. 2002 [74]	Brain, 5	4% PFA perfused brain followed by 12 h postfixation in the same fixative	Crystal placement or solution injection (0.2 μL of 10% DiI in dimethylformamide)	Superior colliculus	7.5 mm	4% PFA 6–12 months 4 °C or room temperature	Commissural connections of the superior colliculus in adults
Tardif E. 2005 [75]	Brain, 5	Tectal plate immersion-fixed in 4% PFA for 12–24 h	Crystal placement in superficial layers	Superior colliculus	6 mm	4% PFA 12–32 months 37 °C	Intrinsic connectivity of the superior colliculus in adults
Tardif E. 2007 [76]	Brain, 7	Tissue blocks fixed in 4% PFA (1 week) or brains perfused (internal carotid and basilar arteries) with 4% PFA followed by postfixation of 12 h by immersion in the same fixative	Crystal placement or solution injection (0.2 μL of 10% DiI in dimethylformamide)	Cortical grey matter	8.8 mm for injected DiI solution	NA 3–54 months 37 °C or room temperature	Intrinsic connectivity of the Broca area in adults
Thal DR. 2008 [77]	Brain, 7	Tissues (10 mm slabs) immersion-fixed 2.6% PFA, 0.8% iodoacetic acid, 0.8% sodium periodate, and 0.1 M d-l Lysine for 5 days	Crystal placement	Entorhinal layers	9.5 mm	2% PFA At least 12 months 37 °C	The temporo-entorhinal connections in adults
Nimchinsky E. 1995 [60]	Brain, 8/NA **	Immersion-fixed brains in 4% PFA for up to 120 h	DiI methanol paste	Cingulum bundle	NA	Phosphate buffer 2–5 months Room temperature	Morphological and anatomical features of spindle neurons in the cingulate cortex of adults
Galuske R. 2000 [41]	Brain, 7	Tissue blocks fixed in solution containing 2.6% PFA, 0.8% iodacetic acid, 0.8% sodiumperiodate, and 0.1 M D-Llysine for 48 h	Crystal placement	Cortical tissue	7 mm	2% PFA 4–6 months 37 °C	Intrinsic connections of primary auditory cortex in adults
Lai HM. 2018 [54]	Brain, 1	Formalin-fixed brain for 5 years	Crystal placement	Deep white matter of cerebellum	3–4 mm	Phosphate buffer 10 days 37 °C	Method for clearing fresh and archived adult brain tissues for 3D visualization
Hildebrand S. 2020 [47]	Brain, 1	Periodate-lysine-paraformaldehyde fixed amygdala blocks for 2–7 days	Crystal placement	Amygdala specimens	0.4 mm	2% PFA 6 months–several years 37 °C	Method for clearing fresh brain tissues for 3D visualization in adults
Meyer B. 2006 [58]	Eye, 9	4% PFA overnight at 4 °C, then retinas transferred in 1% PFA	Crystal placement	Flat-mounted retinas	5 mm	1% PFA >2 months 20 °C	Effect of diabetes and hypertension on the morphology of retinal ganglion cells in adults
Palvidis M. 2003 [63]	Eye, 4	Retinas in 4% PFA overnight at 4 °C	Crystal placement	Flat-mounted retinas	2–5 mm	0.5–1% PFA 4–6 months 4 °C	Effect of glaucoma on the morphology of retinal ganglion cells in adults
Seehaus A. 2012 [70]	Brain, 1	Temporal lobe fixed in 2.6% PFA, 0.8% iodoacetic acid, 0.8% sodium periodate, and 0.1 M D-L-lysine (4 days)	Crystal placement	Surface of temporal cortex block	13 mm	NA 48 months NA	Validate diffusion-weighted MRI using lipophilic tracing in adults
Thanos S. 1991 [78]	Eye, 16/NA *	Retinas in 4% PFA overnight at 4 °C	Crystal placement	Flat-mounted retina	5 mm	2% formalin 4 weeks NA	Morphology of retinal ganglion cells (specimen unknown age)
Burkhalter A. 1993 [33]	Brain, 12	Occipital lobe pieces fixed in 3% paraformaldehyde solution containing 0.1 M lysine-HCI, 0.8% NaIO_4_, and 0.8% iodoacetic acid for 24 h at 4 °C	Crystal placement	V1 or V2 cortex	3 mm (estimate)	Phosphate buffer 1–6 months 21 °C	Development of connections in the visual cortex from fetal to infant period
Cheng G. 2004 [35]	Brain, 8	Tissues immersion-fixed in 4% PFA for 6 months–1 year	Crystal placement	Upper right superior mediastinum or vagal rootlets from groove along the lateral rim of olive	3 mm (estimate)	4% PFA 6–8 weeks 37 °C	Development of vagal nerve afferents/efferents within the tractus solitarius nuclear complex from embryonic to fetal period
deAzevedo L.C. 1997 [37]	Brain, 4	Brains fixed in 4% PFA for 2–3 weeks	Crystal placement	Dorsal half of corpus callosum	NA	4% PFA 4–6 months room temperature	Development of callosal neuron architecture during fetal period
Fitzgibbon T. 1997 [38]	Eye, 32 retinae/NA *	Eyes fixed in 2–4% PFA	Crystal placement	Retina nerve fiber layer	Rarely over 4 mm	2–4% PFA 3–8 weeks 37 °C	Development optic nerve head and retinal nerve fiber layer during fetal period
Hevner R. 2000 [45]	Brain, 4	Brains fixed in 4% PFA/4% Sucrose for 2–7 days, tissue blocks obtained	Crystal placement	Optic tract or optic nerve or optic radiations	NA	4% PFA/4% sucrose 17–45 weeks room temperature	Development of connections in the visual pathway in the mid-gestation fetal brain
Hevner R. 1996 [46]	Brain, 9	Brains fixed in cold 4% PFA/4% sucrose for 2–7 days or 10% formalin at RT	Crystal placement	Entorhinal cortex or dentate gyrus or CA1/Subiculum or CA3	3 mm (estimate)	4% PFA 17–45 weeks Room temperature	Development of connections of hippocampal formation subdivisions by fetal midgestation
Konstantinidou A. 1995 [52]	Spinal cord, 13	4% PFA for 48–72 h	Crystal placement	Spinal cord	NA	Phosphate buffer 1 week 37 °C	Development of fetal dorsal root afferent projections
Loeliger M. 2000 [56]	Brain, 21	Tissue blocks immersed in 4% PFA for at least 3 months	Solution of DiI in 100% ethanol	Cranial nerve ends with brush	15 mm	4% PFA At least 8 months 37 °C	Afferents and efferents of lower cranial nerves in patients with sudden infant syndrome and control infants
Meyer G. 1993 [57]	Brain, 12	Initial fixation in 10% formalin (2 to 24 h) followed by 4% PFA	Crystal placement	Motor and temporal cortex	NA	4% PFA 2–6 weeks 30 °C	Development of fetal layer I in neocortex
Ozturk NC. 2022 [62]	Peripheral and upper and lower cranial nerves, 4	Archival tissue in 4% formalin at room temperature	Crystal or paste placement with micro bendable pin	Peripheral and cranial nerves following microincision	25.11 ± 9.1 mm	4% PFA up to 16 weeks 37 °C	Method for archival fetal tissue DiI tracing
Qu J. 2006 [67]	Brain, 10	Brain fixed in 4% PFA for up to 6 weeks	Crystal placement	Brachium of the superior colliculus or groove between posterior thalamus and rostrolateral rim of crus cerebri	NA	4% PFA Up to 15 weeks 37 °C	Development of the connections between retina and superior colliculus during embryonic and fetal period
Sailaja K. 1994 [68]	Brain, 6	Dissected tissue in 4% PFA or 10% formalin for up to 4 weeks	Crystal placement	Head, body, and tail of caudate nucleus	NA	4% PFA 6 months room temperature	Development of substantia nigra during embryonic and fetal period
Wu L. 2013 [81]	Trigeminal ganglion/ nerves, 5	Intracardial perfusion 10% formalin	Crystal placement	Ophthalmic, maxillary, and mandibular nerves	5 mm	10% formalin 3 months 37 °C	Fetal trigeminal ganglion morphology
Zec N. 1997 [82]	Brain, 23/NA **	Dissected tissues fixed in 4% PFA with 4% sucrose for 48–72 h at 4°C	Crystal placement	Arcuate nucleus, raphe obscurus nucleus, pyramid, corticospinal tract	19–28 mm	4% PFA with 4% sucrose 7–15.5 months room temperature	Fetal arcuate and caudal raphe nuclei connectivity
Zec N. 2001 [83]	Brain, 9	Dissected tissues fixed in 4% PFA with 4% sucrose at 4 °C for 24 h, tissue blocks fixed for additional 24–48 h	Crystal placement	Nucleus Paragigantocellularis lateralis	20–28 mm	4% PFA with 4% sucrose 8.5–15.5 months room temperature	Fetal paragigantocellularis lateralis nucelus connectivity
Zec N. 2003 [84]	Brain, 10	Dissected tissues fixed in 4% PFA with 4% sucrose at 4 °C for 24 h, tissue blocks fixed for additional 24–48 h	Crystal placement	Nucleus of the solitary tract	NA	4% PFA with 4% sucrose 6–16 months room temperature	Fetal nucleus tractus solitarius connectivity
Das S. 2019 [36]	Brain, 13	Tissue blocks fixed in 4% cold paraformaldehyde for 1 h, followed by 4% PFA/0.125% glutaraldehyde for 24 h at 4 °C, 350 µm sections with vibratome	Crystal placement	CA1 sub-region and supra-pyramidal blade of the dentate gyrus	NA	Phosphate buffer 72 h 37 °C	Method for the study of pre- and postsynaptic elements in hippocampus across the lifespan (4 months to 71 years)
Burkhalter A. 1993 [31]	Brain, 6	Brains fixed 2.6% paraformaldehyde solution containing 0.1 M lysine-HCI, 0.8% NaIO_4_, and 0.8% iodoacetic acid for 24 h at 4 °C. Blocked into 5 mm slabs	Crystal placement	V1 or V2 cortex	3 mm (estimate)	Phosphate buffer 1–6 months 21 °C	Development of connections between V1 and V2 visual cortical areas from fetal to infant period
Hayaran A. 1992 [43]	Brain, 6	Dissected tissues fixed in 4% PFA for 4–6 weeks	Crystal placement	Olive or cerebellar dentate nucleus	NA	4% PFA 12–16 weeks room temperature	Method for polyacrylamide infiltration and embedding of DiI-stained fetal cerebellum
Bystron I. 2005 [34]	Brain, 21/NA *	Whole heads in 4% PBS for 7–24 h	Crystal placement	Parts of telencephalon or diencephalon	NA	Phosphate-buffered saline 3–4 weeks NA	Embryonic human forebrain early axonal outgrowth
Hens J. 2001 [44]	Jejunum, 4	Unfixed in oxygenated Krebs solution	Crystal placement	Intestinal villus	“Several mm”	Culture medium 4–5 days 37 °C	Enteric neurons innervating the mucosa of the small intestine during infant life
Abel R.M. 1998 [26]	Pylorus, 14	Specimens fixed in 2% formalin	Crystal placement	Severed surface of the vagus nerve or surface of pylorus (control)	NA	Phosphate buffer 6 months Room temperature	Fetal pylorus vagal innervation
Humenick A. 2019 [49]	Colon, 21	Unfixed (post-DiI fixed in Zamboni’s for 24–48 h)	Tracer-covered glass beads	Serosal surface (fills from inter-tenial muscle) or incision of tenial muscle (fills of tenia)	12 mm	Culture medium 4–5 days 37 °C	Innervation of longitudinal muscle of colon by motor neurons in adults
Humenick A. 2020 [50]	Colon, 36/NA **	Unfixed (post-DiI fixed in Zamboni’s for 24–48 h)	Tracer-covered glass beads	Surface of the circular muscle or ganglia or longitudinally running internodal strands of the myenteric plexus	70 mm	Culture medium 4–5 days 37 °C	Characterization of interneurons in myenteric plexus of colon in adults
Swift M. 2005 [72]	Peripheral nerves, 6	10% formalin-fixed cadavers	Solution (5–10 μL of 1 mg/mL in ethanol) Or paste	Median and ulnar nerves or cutaneous nerves	Electrical fields for 48 h: 53.7 ± 1.66 mm, controls: 8.1 ± 0.52 mm	Mineral oil immersion (of silicone gel coated samples) for up to 10 days Room temperature	Method to accelerate tracer diffusion and increase tracing distance in peripheral nerve tissue in adults
Porter A. 1997 [64]	Colon, 13	Unfixed (post-DiI fixed with modified Zamboni’s for 16–24 h at 4 °C)	Tracer-covered glass beads	Circular muscle	20 mm	Culture medium 5 days 37 °C	Motor neurons of the circular muscle of the human colon in adults
Porter A. 1999 [65]	Colon, 11	Unfixed (post-DiI fixed with modified Zamboni’s for 16–24 h at 4 °C	Tracer-covered glass beads	Mucosa or submucosa, or circular muscle	9.3 mm	Culture medium 5 days 37 °C	Connections within and between the mucosa, submucosa, and muscle layer of colon in adults
Porter A. 2002 [66]	Colon, 8	Unfixed (post-DiI fixed with modified Zamboni’s for 16–24 h at 4 °C	Tracer-covered glass beads	Incision of myenteric plexus	33 mm	Culture medium 5 days 37 °C	Cholinergic and nitrergic neurons in colon (myenteric plexus) in adults
Wattchow D. 1995 [79]	Intestine, 28	Unfixed (post-DiI fixed with modified Zamboni’s for 16–24 h at 4 °C)	Tracer-covered glass beads	Mucosa or submucosa, or circular or longitudinal muscle layer or myenteric plexus	68 mm	Culture medium 3–5 days 37 °C	Projections and morphology of myenteric neurons in adults
Wattchow D. 1997 [80]	Colon, 13	Unfixed (post-DiI fixed with modified Zamboni’s for 16–24 h at 4 °C)	Tracer-covered glass beads	Circular muscle or incision through the myenteric plexus	30 mm	Culture medium 5 days 37 °C	Polarity of neurochemically defined myenteric neurons in the human colon in adults

Abbreviations: DMSO: Dimethylsulfoxide; PFA: Paraformaldehyde; NA: Not available; LOHN: Leber hereditary optic neuropathy; PBS: Phosphate-buffered saline; HSV-1: Herpes simplex virus 1; CA: cornu Ammonis. * Total number of specimens. ** Total number of subjects.

## Data Availability

Data are available from the corresponding author upon reasonable request.

## Appendix A

**Table A1:** Algorithms used for database search.

Database: PubMed Date of last search: 18 March 2024 Number of results: 695 Algorithm used: ((“Di-I”[All Fields] OR “DiI”[All Fields] OR “1 1 dioctadecyl 3 3 3 3 tetramethylindocarbocyanine perchlorate”[All Fields] OR “diic18 3”[All Fields] OR “D 282”[All Fields] OR “FAST DiI”[All Fields]) AND ((“neuron*”[All Fields] OR “axon*”[All Fields] OR “nerv*”[All Fields] OR (“brain”[MeSH Terms] OR “brain”[All Fields] OR “brains”[All Fields]) OR (“spine”[MeSH Terms] OR “spine”[All Fields] OR “spines”[All Fields]) OR “plexus”[All Fields]) AND “trac*”[All Fields])) Filter: English language
Database: Scopus Date of last search: 18 March 2024 Number of results: 766 Algorithm used: ((TITLE-ABS-KEY (“Di-I” OR “DiI” OR “1 1 dioctadecyl 3 3 3 3 tetramethylindocarbocyanine perchlorate” OR “diic18 3” OR “D 282” OR “FAST DiI”)) AND (TITLE-ABS-KEY (“neuron*” OR “axon*” OR “nerv*” OR “brain*” OR “spine*” OR “plexus”) AND TITLE-ABS-KEY (“trac*”))) AND (LIMIT-TO (LANGUAGE, “English”))
Database: Web of Science Date of last search: 18 March 2024 Number of results: 661 Algorithm used: ((ALL = (“Di-I” OR “DiI” OR “1 1 dioctadecyl 3 3 3 3 tetramethylindocarbocyanine perchlorate” OR “diic18 3” OR “D 282” OR “FAST DiI”)) AND ALL = (((“neuron*” OR “axon*” OR “nerv*” OR “brain*” OR “spine*” OR “plexus”) AND “trac*”))) Filter: English language
